# Gene expression profiling during adventitious root formation in carnation stem cuttings

**DOI:** 10.1186/s12864-015-2003-5

**Published:** 2015-10-14

**Authors:** Carlos Villacorta-Martín, Ana Belén Sánchez-García, Joan Villanova, Antonio Cano, Miranda van de Rhee, Jorn de Haan, Manuel Acosta, Paul Passarinho, José Manuel Pérez-Pérez

**Affiliations:** Genetwister Technologies B.V., P.O. Box 193, NL6700 AD Wageningen, The Netherlands; Instituto de Bioingeniería, Universidad Miguel Hernández, 03202, Elche, Alicante, Spain; Departamento de Biología Vegetal (Fisiología Vegetal), Universidad de Murcia, Murcia, Spain

**Keywords:** RNA-Seq, Time-series analysis, Differential expression profiling, Regulatory network analysis, *Dianthus caryophyllus*

## Abstract

**Background:**

Adventitious root (AR) formation is a critical step in vegetative propagation of most ornamental plants, such as carnation. AR formation from stem cuttings is usually divided into several stages according to physiological and metabolic markers. Auxin is often applied exogenously to promote the development of ARs on stem cuttings of difficult-to-root genotypes.

**Results:**

By whole transcriptome sequencing, we identified the genes involved in AR formation in carnation cuttings and in response to exogenous auxin. Their expression profiles have been analysed through RNA-Seq during a time-course experiment in the stem cutting base of two cultivars with contrasting efficiencies of AR formation. We explored the kinetics of root primordia formation in these two cultivars and in response to exogenously-applied auxin through detailed histological and physiological analyses.

**Conclusions:**

Our results provide, for the first time, a number of molecular, histological and physiological markers that characterize the different stages of AR formation in this species and that could be used to monitor adventitious rooting on a wide collection of carnation germplasm with the aim to identify the best-rooting cultivars for breeding purposes.

**Electronic supplementary material:**

The online version of this article (doi:10.1186/s12864-015-2003-5) contains supplementary material, which is available to authorized users.

## Background

In horticulture and forestry, vegetative propagation is widely used for the multiplication of plants with optimal phenotypes obtained in breeding programs or selected from natural populations. Adventitious root (AR) formation is a critical step in vegetative propagation: substantial losses can occur because cuttings do not form roots or they form poor quality root systems. A conservative estimation quantifies the burden of inadequate rooting treatments on US $50 million per year only in The Netherlands.

ARs are distinct from lateral roots in that they form from any tissue that is not a root, such as leaves and stems, naturally or in response to altered environments [1, 2]. AR formation from stem cuttings is usually divided into several stages according to physiological and metabolic markers: *i)* dedifferentiation, during which cells become competent to respond to the rhizogenic signal (auxin), *ii)* cell division (or induction phase), and *iii)* root primordia outgrowth from the stem [3]. Several plant hormones are known to control AR formation, of which auxin is a central player [4]. Auxin is often applied exogenously to promote the development of ARs on stem cuttings of difficult-to-root genotypes [1, 3]. In many species, high auxin levels in the basal region of the cuttings are required for the competent cells in the cambium to resume proliferation and to start the root-specific developmental program [5, 6]. Consistently with a positive role for auxin in AR formation, Arabidopsis mutants overproducing auxin spontaneously develop ARs on the hypocotyl [7–9]. Auxin and cytokinins are known to play a crucial role in many aspects of plant development, often acting antagonistically. A negative role for cytokinins in AR formation has been proposed as mutants defective in cytokinin biosynthesis or perception displayed increased production of ARs, whereas enhanced cytokinin biosynthesis has the opposite effect [10–12]. Moreover, interrelationships between auxin and carbohydrate metabolism during adventitious rooting have been investigated by the application of exogenous auxins and by monitoring of carbohydrate levels, carbon translocation and activities of key enzymes involved in sugar metabolism in the rooting zone [13–15].

Various molecular approaches have been employed to study AR development in Arabidopsis and other model plants [1]. In Arabidopsis, it was shown that the balance between AUXIN RESPONSE FACTOR17 (ARF17), a negative regulator of adventitious rooting, and ARF6 and ARF8, positive regulators of AR formation, as well as the maintenance of the homeostasis of their regulatory microRNAs (miRNAs), plays a critical role in AR formation [16, 17]. Additionally, the proteomic analysis of mutants affected in AR formation led to the identification of 11 proteins, including three auxin-inducible GRETCHEN HAGEN (GH3)-like proteins, whose expression was altered [18]. In turn, these GH3-like proteins are required for fine-tuning AR initiation in the hypocotyl, through modulating jasmonic acid homeostasis [19]. These results suggest that the early stages of AR formation need to be tightly regulated at the physiological and the genetic level and that improving rooting performance of economically important genotypes requires identifying the molecular components of the hormonal crosstalk that regulates AR formation in non-model species. As an alternative strategy to identify genes involved in AR formation, a number of studies have been conducted to characterize the gene expression profiles in the stem cutting base of different species during rooting [13, 20–25]. Based on these studies, some of the molecular events occurring during AR formation have been outlined. In our study, we aimed to characterize gene expression and functional changes occurring in the stem cutting base during the early stages of adventitious rooting in two carnation cultivars, *2003 R 8* and *2101*–*02 MFR*, which have been selected because of their contrasting rooting performance [26]. Our results will allow the identification of the genes involved in AR formation in this species, which will contribute to our basic understanding of the molecular events leading to this complex developmental response.

## Methods

### Plant material and growth conditions

**Fig. 1:**
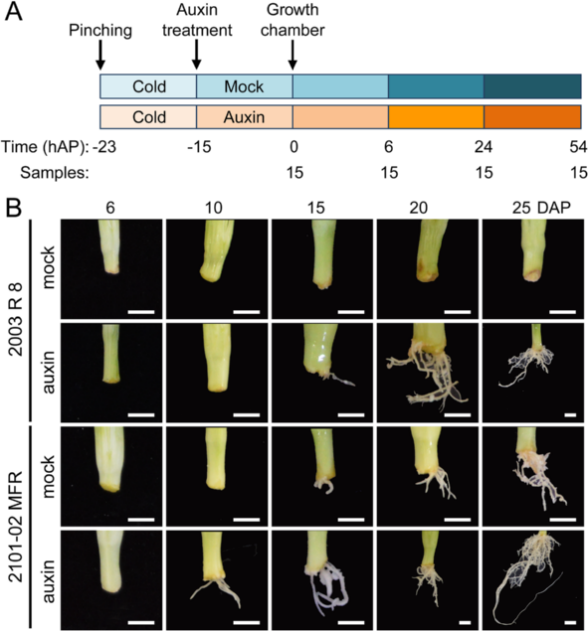
Experimental design and time-series analysis of AR formation in two carnation cultivars. **a** Schematic representation of the experimental design followed in our time-course experiment. hAP: hours after planting. **b** Representative images of the basal stem of carnation cuttings of the *2003 R 8* and *2101*–*02 MFR* cultivars between 6 and 25 days after planting (DAP). Scale bars: 5 mm

Stem cuttings were pinched from several mother plants of the *2003 R 8* and *2101*–*02 MFR* cultivars by skilled operators at noon on 2^nd^ December 2013 (−23 h). About 500 stem cuttings of each cultivar were wrapped in plastic bags after pinching and were sent refrigerated and in complete darkness to the laboratory (−15 h). Next, the stem cutting bases were submerged for 15 h in a 100 ml-water solution containing either mock or an auxin cocktail (1.5 μM indole-3-butyric acid [IBA; Duchefa, The Netherlands] and 1 μM α-naphtalene acetic acid [NAA; Duchefa, The Netherlands]). After the treatment, the cuttings were individually planted in 70-well trays containing moistened perlite plugs (4.5 × 4.5 × 4.5 cm; 90 cm^3^), and their basal regions were collected at 0, 6, 24 and 54 h after planting (hAP) in a walk-in growth chamber that was set at 22 ± 2°C, 70 % relative humidity and under continuous fluorescent light with an average photosynthetic photon flux density of 40 μmol m^−2^ s^−1^. Three biological replicates, each consisting of fifteen stem cutting bases (~5 mm long), were collected per cultivar, treatment and time point, and were immediately frozen in liquid N_2_. To minimize variation due to subtle environmental differences within the growth chamber, an incomplete block experimental design was used. The experimental design used for sample collection is shown in Fig. 1a.

### RNA isolation, library construction and Illumina sequencing

For each sample, total RNA from ~120 mg of powdered stem cutting base tissue that was kept at −65 °C was extracted using Spectrum™ Plant Total RNA Kit (Sigma-Aldrich, USA). The RNA integrity was confirmed using the 2100 Bioanalyzer (Agilent Technologies, USA). External RNA Controls Consortium RNA Spike-In mixes (Life technologies, USA) were used to assess the sensitivity and dynamic range of the experiment. The samples were prepared for sequencing using the TruSeq RNA Sample Preparation Kit v2 (Illumina, USA) . Illumina 100 bp paired-end sequencing on the HiSeq2000 was carried out by Macrogen, Korea. The raw Illumina reads were pre-processed using our in-house quality control pipeline. The 3’ends with a quality score below 20 were trimmed.

### Reference genome: feature re-annotation and functional annotation

We used the carnation reference genome assembly released by [27]. We extended the available gene prediction using transcript sequence evidence. To this end, we assembled a comprehensive transcriptome with RNA sequencing (RNA-Seq) data comprising the sum of eight different tissues and cultivars. Each of the transcriptome assemblies was done using a genome-guided hybrid approach with Trinity [28]. Next, we leveraged this information by first aligning the transcripts to the annotated genome. The best alignments between transcript and genome annotation (those spanning at least 90 % of the transcript length) were selected and subsequently clustered in groups based on a minimum overlap of 30 % between alignments. These clusters were used as evidence to update the existing feature annotation with PASA [29]. To obtain a functional annotation, open reading frames (ORFs) were inferred from the updated, evidence-based gene models using Transdecoder [29]. We then blasted these ORFs against a database comprising all the complete proteins from core-eudicots with a Gene Ontology (GO) [30] annotation (approximately 200,000 proteins). The use of a relatively small and highly informative set of proteins as a database increases power (smaller search-space, smaller e-values) and minimizes the chance for noisy alignments with non-homologous or non-annotated proteins. The ORFs were also compared to model profiles from the Pfam domain database [31] using HMMER [32]. The output from these sequence comparisons was integrated using ARGOT2 [33] to assign one (or several) GO annotation to each ORF. Beside this functional annotation, and in order to include information that is mainly available in model species, we mapped carnation genes to their putative *Arabidopsis thaliana* (Arabidopsis) orthologues from the The Arabidopsis Information Resource (TAIR) database [34] by means of: *i)* reciprocal best-hits between carnation and Arabidopsis proteomes, and *ii)* one-way best-hits (OBH) of carnation to the Arabidopsis proteome.

### Exploratory data analysis and differential expression tests

Prior to the differential expression analysis with the DESeq2 package [35], we assessed the overall similarity between samples in order to check that it fitted the expectation from the experimental design. We calculated the Euclidean distance between samples using regularized-log transformed expression values to avoid that a few highly variable genes dominated the distance measure. We also used principal-component analysis (PCA) to examine the similarity between samples according to the components that explain most of the variance in the data as shown in Additional file 1: Figure S1.

Using the DESeq2 package we fitted generalized linear models of gene expression. The significance of the coefficient of the fitted models was inferred using a Wald test. To increase power, we filtered out genes with zero counts in all the samples, which reduces the burden of a strong multiple test correction [36]. This reduced the data to 37,849 genes. Biological replicates were considered for each time point, as previously described [37]. Functional enrichment was tested with topGO [38] for the lists of resulting differentially expressed genes (DEG).

### Gene expression analysis by quantitative reverse-transcription PCR

The selection of candidate genes for their experimental validation by quantitative reverse-transcription PCR (qRT-PCR) was based on the following criteria: *i)* high relative expression level in the RNA-Seq experiment at 0 hAP, *ii)* the function of its putative Arabidopsis ortholog was related to root growth and development, and *iii)* a dynamic expression range across the evaluated period. Six genes fulfilling these criteria were chosen for qRT-PCR analysis: *Dca5879*, *Dca23172*, *Dca29160*, *Dca30890*, *Dca40234* and *Dca43825*. For primer design, small amplicons (90 to 140 bp) were chosen within the first third of the cDNA sequences. Whenever possible, forward and reverse primers bind to different exons and the reverse primer was designed to hybridize with two consecutive exons to avoid amplification of genomic DNA.

The first strand cDNA was synthesized from 1 μg of purified RNA using the iScript Reverse Transcription Supermix for RT-qPCR (Bio-Rad, USA). The resulting cDNA was diluted by adding 40 μl of sterile distilled water. Fourteen μl reactions were prepared with 7 μl of the SsoAdvanced™ Universal SYBR® Green Supermix (Bio-Rad), 5 μM of specific primer pairs (Additional file 2: Table S1) and 1 μl of cDNA. PCR amplifications were carried out in 96-well optical reaction plates on a Step One Plus Real-Time PCR System (Applied Biosystems, USA). Two independent RNA isolates and three technical replicates were used per cultivar, treatment and time point assayed. The thermal cycling program started with a step of 10 s at 95 °C, followed by 40 cycles (15 s at 95 °C and 60 s at 60 °C), and the melt curve (from 60°C to 95°C, with increments of 0.3°C every 5 s). Dissociation kinetics and agarose gel loading of the amplified products confirmed their specificity.

Primer pair validation and relative quantification of gene expression levels were performed by using the 2^-∆∆CT^ method [39]. The *Dca17200* gene (the putative homolog of the Arabidopsis housekeeping gene *ACTIN2*; *AT3G18780*) was chosen for normalization of the assayed genes as its expression was constant among the different cultivars, treatments and time points studied. All samples were compared to the expression level of the control treatment (mock) at the zero-time point (0 hAP). The average of fold-change values were used for graphic representation.

### Time-course analysis

To cluster genes according to their time-course profile, we reformatted cross-sectional data where each sample corresponds to cuttings from different plants (*i.e.,* destructive sampling) as longitudinal data. To this purpose, the normalized counts of replicated samples at different time points were paired, producing complete time courses. To handle the missing data of one of the mock replicates at 0 hAP in the cultivar *2003 R 8*, missing values were imputed averaging the normalized counts from the other two replicates at the same time point and cultivar. Gene clustering and GO enrichment analysis within clusters was performed in STEM [40] using default parameters (STEM Clustering Method). In order to increase the signal-to-noise ratio, we filtered out genes with a log expression difference across time points smaller than 1.25 and correlation between replicates smaller than 0.75.

### Light microscopy

For each cultivar and treatment, ~5 mm long segments from the base of the stem cuttings were sectioned at different time points (0, 6, 24 and 54 hAP). Samples were fixed in a FAA/Triton solution (1.85 % v/v formaldehyde, 45 % ethanol, 5% acetic acid, and 1 % Triton X-100) for 8 h on a light vacuum (400 mbar) until the tissue sank. Samples were then kept in the FAA/Triton solution for 3 days at 4 °C. The fixed tissue was rinsed 3 times in 0.1 M sodium phosphate buffer (pH 7.2) before dehydrating in a graded ethanol series (70, 80, 90 and 96 % ethanol, 60 min each step). Dehydrated samples were then embedded in Technovit 7100 resin (Heraeus Kulzer GmbH, Germany) according to the manufacturer’s instructions with slight modifications, as follows. Samples were immersed in the pre-infiltration solution (50 % v/v resin and 50 % ethanol) for 2.5 h. Then, stem cutting samples remained 4 h in the infiltration solution on a light vacuum at room temperature and polymerized for 20 h at 4 °C. Thin sections of 7 μm-thickness were cut using a tungsten microtom knife (MICROM International GmbH, Germany) on a HS 350 S rotary microtome (MICROM International GmbH). Sections were stained either with 0.05 % weight/volume (W/V) toluidine blue (Sigma-Aldrich) or 0.05 % W/V ruthenium red (Sigma-Aldrich) in water and mounted in Eukitt (Chem-Lab NV, Belgium). Samples were observed using a bright-field Motic BA210 microscope (Motic Spain, Spain) and selected images were captured with a built-in Moticam 580INT documentation station (Motic Spain) and processed with Adobe Photoshop CS3.

### Phytohormone extraction and analysis

Phytohormones were extracted and analysed according to [41]. Briefly, ~100 mg of frozen tissue from the same batches used for the RNA-Seq experiment were extracted twice with 1 ml of methanol/water 80 %, centrifuged at 20,000 *g* for 15 min. at 4 °C, the supernatant was passed through a C18 cartridge, and the samples were collected in a 5-ml tube for speed-Vac evaporation to dryness. The residue was resuspended in 1 ml methanol/water 20 %. Ten μl of filtrated extract were injected in a U-HPLC-MS system consisting of an Accela Series U-HPLC (ThermoFisher Scientific, USA) coupled to an Exactive mass spectrometer (ThermoFisher Scientific) using a heated electrospray ionization interface. Mass spectra were obtained using the Xcalibur software version 2.2 (ThermoFisher Scientific). For quantification of the plant hormones, calibration curves were constructed for each analysed component (1, 10, 50, and 100 μg l^−1^) and corrected for 10 μg l^−1^ deuterated internal standards. Recovery percentages ranged between 92 and 95 %.

### Transcription factor analysis

To find out which transcription factor (TF) families are likely to play a more important role along the experimental process, we analysed their enrichment among genes annotated with the function “sequence-specific DNA binding transcription factor activity” (GO:0003700). In correspondence with the filtering criteria for the time-course analysis, we excluded genes with a log expression difference across time smaller than 1.25 and correlation between replicates smaller than 0.75. Carnation genes with predicted transcription factor activity were classified in families via their putative Arabidopsis orthologs, based on the OBH method (see functional annotation section). The family classification of these orthologs was obtained from the Database of Arabidopsis Transcription Factors [42]. Genes mapped to TF families were further categorised as upregulated or downregulated according to their profiles in the time-course analysis at 54 hAP. For each category, a Fisher exact test was done to assess significant enrichment. P-values were adjusted for multiple testing (Benjamini-Hochberg).

## Results

### Sequencing and transcriptome assembly supports the discovery of novel genes expressed in the stem cutting base

In a recent study [26] we characterized AR formation in a collection of 10 carnation cultivars. The *2003 R 8* and the *2101*–*02 MFR* cultivars have been chosen for further studies due to their differences in rooting performance and in their differential response to a mild auxin treatment during rooting (Fig. 1b). The bad-rooting behaviour of the *2003 R 8* cultivar, which was mostly caused by a delay in AR initiation, was partially restored by exogenous auxin application.

Several cDNA libraries prepared from stem cutting bases of mock-treated and auxin-treated samples at particular time points during adventitious rooting (0, 6, 24 and 54 hAP) were sequenced (see Methods; Fig. 1a). As a result, 3,683 million of raw reads were obtained. The amount of expression data generated in our study had the potential to transform the boundaries and extent of previous feature annotations in the carnation genome [27]. Genome-guided assemblies were performed with the purpose of serving as sequence evidence for a genome re-annotation. Thus, our updated, evidence-based annotation comprised 59,396 transcripts, corresponding to 57,641 genes, with an average length of 2,856 bp (Table 1). We were able to merge exons from genes that had been previously predicted as separate coding sequences [27]; these merges resulted in a more complete or contiguous annotation for 394 genes and their corresponding transcripts (see Additional file 3). In order to quantify the improvements of the new annotation in relation to the former annotation, we compared the proportion of reads mapping to each of them and also generated a number of descriptive statistics (Table 1).

**Table 1:** Comparison between the previously published annotation and the updated genome annotations

Feature	Yagi et al. [27]	Evidence-based annotation
Gene count	56,137	57,641
Transcript count	56,382	59,396
Average transcript length	2,742	2,856
Median transcript length	2,065	2,125

### Time-dependent comparison of the auxin treatment identifies 1,286 differential expressed genes (DEGs) in response to the auxin stimulus

We then tested the effects in gene expression of factors like cultivar, treatment and time point using different models and contrasts (Table 2). Three questions were addressed: *i)* for which genes does the cultivar factor have a significant effect? (Table 2, test 1), *ii)* which genes change their expression over each pair of time points? (while accounting for cultivar-specific effects; Table 2, test 2–13) and *iii)* how does the auxin treatment affect gene expression distinctively at each time point for each cultivar? (Table 2, test 14–21). To reduce the complexity of the model, in this last case, we made subsets of samples belonging to each cultivar and estimated the parameters separately.

**Table 2:** Differential expression tests

Test	Data subset	Model formula	Contrast tested	DEGs
1	All	C + Ti + C : Ti	2003 R 8 *vs.* 2101–02 MFR	23,029
2	All	C + Ti + C : Ti	2003 R 8:Ti 1 *vs.*2003 R 8:Ti 2	3,820
3	All	C + Ti + C : Ti	2003 R 8:Ti 1 *vs.* 2003 R 8:Ti 3	4,645
4	All	C + Ti + C : Ti	2003 R 8:Ti 1 *vs.* 2003 R 8:Ti 4	5,880
5	All	C + Ti + C : Ti	2003 R 8:Ti 2 *vs.* 2003 R 8:Ti 3	2,828
6	All	C + Ti + C : Ti	2003 R 8:Ti 2 *vs.* 2003 R 8:Ti 4	2,983
7	All	C + Ti + C : Ti	2003 R 8:Ti 3 *vs.* 2003 R 8:Ti 4	690
8	All	C + Ti + C : Ti	2101-02 MFR:Ti 1 *vs.* 2101–02 MFR:Ti 2	11,536
9	All	C + Ti + C : Ti	2101-02 MFR:Ti 1 *vs.* 2101–02 MFR:Ti 3	12,694
10	All	C + Ti + C : Ti	2101-02 MFR:Ti 1 *vs.* 2101–02 MFR:Ti 4	12,129
11	All	C + Ti + C : Ti	2101-02 MFR:Ti 2 *vs.* 2101–02 MFR:Ti 3	9,336
12	All	C + Ti + C : Ti	2101-02 MFR:Ti 2 *vs.* 2101–02 MFR:Ti 4	9,125
13	All	C + Ti + C : Ti	2101-02 MFR:Ti 3 *vs.* 2101–02 MFR:Ti 4	3,430
14	2003 R 8	Ti + Ti : Tr	Ti 1:Aux *vs.* Ti 1:Mock	86
15	2003 R 8	Ti + Ti : Tr	Ti 2:Aux *vs.* Ti 2:Mock	1
16	2003 R 8	Ti + Ti : Tr	Ti 3:Aux *vs.* Ti 3:Mock	
17	2003 R 8	Ti + Ti : Tr	Ti 4:Aux *vs.* Ti 4:Mock	6
18	2101-02 MFR	Ti + Ti : Tr	Ti 1:Aux *vs.* Ti 1:Mock	1,188
19	2101-02 MFR	Ti + Ti : Tr	Ti 2:Aux *vs.* Ti 2:Mock	21
20	2101-02 MFR	Ti + Ti : Tr	Ti 3:Aux *vs.* Ti 3:Mock	1
21	2101-02 MFR	Ti + Ti : Tr	Ti 4:Aux *vs.* Ti 4:Mock	

Datasets were fitted to the corresponding formula (C = cultivar, Ti = time point, Tr = treatment; additive effects are represented by “+”; “:” represents interaction). After model fitting, selected factors were tested (Ti 1 = 0 hAP, Ti 2 = 6 hAP, Ti 3 = 24 hAP, Ti 4 = 54 hAP). DEG: Differential expressed genes; *P-adj*. < 0.05 (Benjamini-Hochberg correction)

In all cases, expression models were fitted to our time-course study by treating each time point as a different “experimental group”, even though the inherent ordering and spacing provided by time points is ignored then.

To investigate the dependencies between treatments and time, explicitly addressing the question of when a gene is differentially expressed, we modeled the interaction between time and treatment as a covariate. Of 57,641 genes, we filtered out genes that were not expressed (0 counts), resulting in a total of 37,936 genes tested for the subset of cultivar *2101*–*02 MFR* and 37,849 for the cultivar *2003 R 8* subset. The factorial analysis identified a total of 1,286 distinct genes as differentially expressed between auxin-induced and control cuttings over different time points (Table 2, test 14–21). Most auxin-related expression changes took place in the initial time points (0 hAP *vs*. 6 hAP). Among them, DEGs of *2101*–*02 MFR* were associated (Fisher exact test) to functions like photosynthesis (GO:0015979; *P* <0.001) and chlorophyll binding (GO:0016168; *P* <0.001). As for the same comparison in the cultivar with poor rooting performance, *2003 R 8*, the functions associated to DEG were translational initiation factor activity (GO:0003743, *P* = 0.0025), and negative regulation of signal transduction (GO:0009968, *P* = 0.0024), among others.

### Clustering of time-course expression profiles reveals co-expression of functionally related genes

**Fig. 2:**
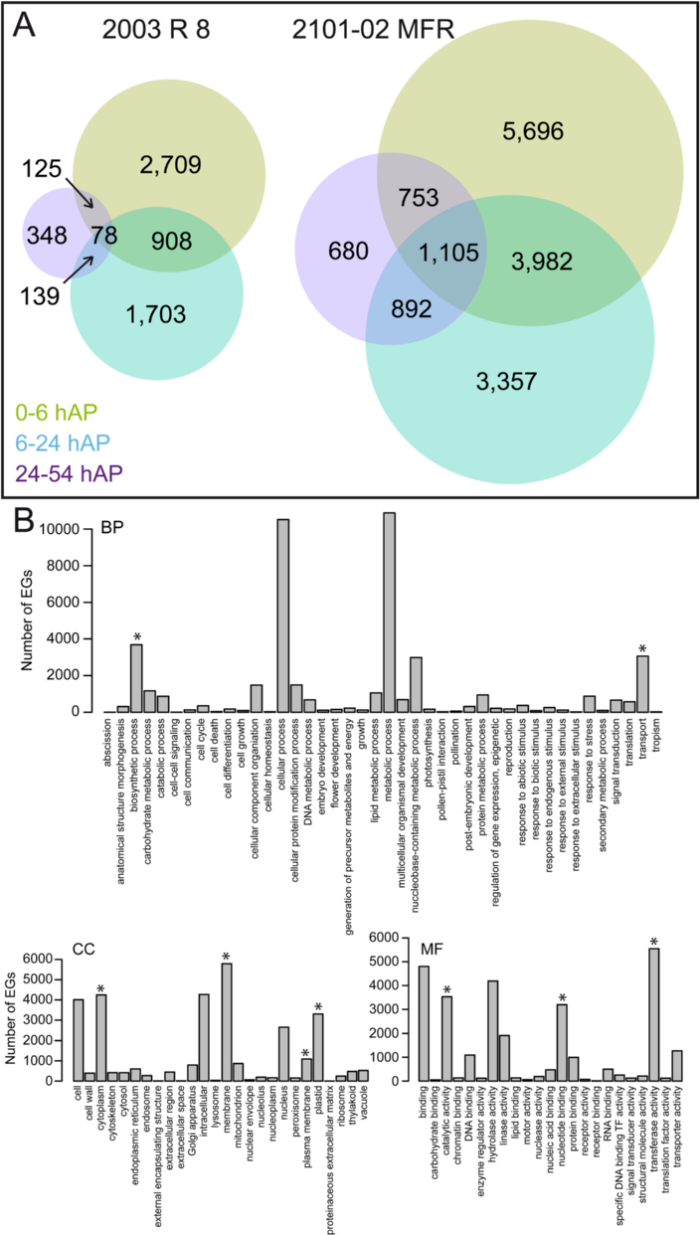
Differentially expressed genes (DEGs) over time during AR formation. **a** Venn diagram illustrating DEGs (*P* < 0.05, Benjamini-Hochberg correction) in the stem cutting base of the *2003 R 8* and *2101*–*02 MFR* cultivars over time. **b** Gene ontology (GO) classification of genes whose expression changes more than 1.25 log-fold over the time-course with respect to biological process (BP), cellular component (CC) and molecular function (MF). Asterisks indicate significant enrichment of genes (P < 0.05)

To get some insight into the specific pathways regulated at different time points during AR formation in the two cultivars studied, we performed a GO-enrichment analysis for the sets of DEGs shown in Fig. 2a. In the *2003 R 8* cultivar, the GO categories “protein amino acid phosphorylation” (GO:0006468; 105 genes; *P* < 0.001) and “transmembrane transport” (GO:0055085; 50 genes; *P* < 0.001) were specifically and significantly enriched at 6–24 and 24–54 hAP, respectively. Interestingly, the “auxin-activated signalling pathway” (GO:0009734; 11 genes; *P* < 0.001) category was found significantly enriched among DEGs shared between 0–24 hAP in this cultivar. Conversely, in the *2101*–*02 MFR* cultivar, the GO category “hormone-mediated signalling pathway” (GO:0009755; 58 genes; *P* < 0.001) was specifically enriched at 0–6 hAP. Moreover, the “auxin-activated signalling pathway” (GO:0009734; 34 genes; *P* < 0.001) and “cell cycle” (GO:0007049; 79 genes; *P* < 0.001) categories were found significantly enriched for DEGs shared between 0 and 24 hAP. The GO categories “glucose catabolic process” (GO: 0006007; 41 genes; *P* < 0.001), and “cellulose biosynthetic process” (GO:0030244; 22 genes; *P* = 0.002) were significantly enriched specifically at 6–24 hAP, whereas at 24–54 hAP the most significant GO-enrichment was found for genes assigned to the “response to stress” (GO:0006950; 46 genes; *P* = 0.007) category.

To obtain a more general view of the functions involved in the early stages of AR formation, we transformed the GO functional annotation into its cut-down version (Plant GO slim) for the 14,554 DEGs in the *2101*–*02 MFR* cultivar between 0 hAP and 6 hAP (Fig. 2b). The Biological Process (BP) classification of DEGs highlighted a significant enrichment (*P* < 0.001) for the following GO categories: “biosynthetic process” (GO:0009058) and “transport” (GO:0006810). Among the Cellular Component (CC) categories, “plastid” (GO:0009536) and “plasma membrane” (GO:0005886) were the most significantly enriched ones. In terms of Molecular Function (MF), a significant enrichment was found for genes at the categories “transferase activity” (GO:0016740) and “nucleotide binding” (GO:0000166). These results indicated that large expression changes are taking place at transcriptome level in the stem cutting base during the initial stages of AR formation in this cultivar.

### Validation of expression of some of the genes detected during AR formation

**Fig. 3:**
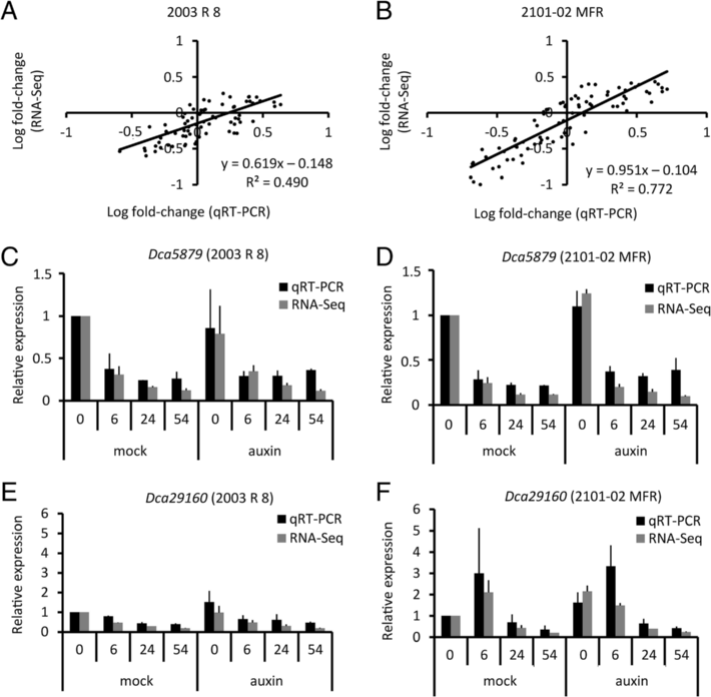
qRT-PCR validation of RNA-Seq results. **a**-**b** Correlation analysis of qRT-PCR and RNA-Seq data from selected genes in the *2003 R 8* (**a**) and *2101*–*02 MFR* (**b**) cultivars. Each dot represents the relative expression data for a given gene and a given sample. **c**-**f** Bars represent the mean of the relative expression level of qRT-PCR (black) or RNA-Seq (grey) data relative to mock-treated samples at 0 hAP. Error bars indicate the standard deviation for the mean data shown

The reliability of our transcriptome profiling dataset was validated by examining the expression of selected genes by using qRT-PCR and by comparing them to the normalized data obtained in the RNA-Seq analysis (see Methods). We found highly significant and positive correlations between qRT-PCR and RNA-Seq results for both cultivars in all time points and treatments (Fig. 3a-b). Additional statistical analysis revealed that the variation observed between qRT-PCR and RNA-Seq results depended largely on the expression levels of the studied genes (Additional file 4: Figure S2). Thus, for genes with very low or very high numbers of RNA-Seq reads, the qRT-PCR validation was less accurate. Representative examples of the results obtained for genes with contrasting expression profiles are shown in Fig. 3c-f. While the expression levels of *Dca5879* were only varying over time (Fig. 3c-d), those of *Dca29160* were also depending on cultivar and the auxin treatment (Fig. 3e-f).

### Gene set enrichment analysis in the *2003 R 8* cultivar

**Fig. 4:**
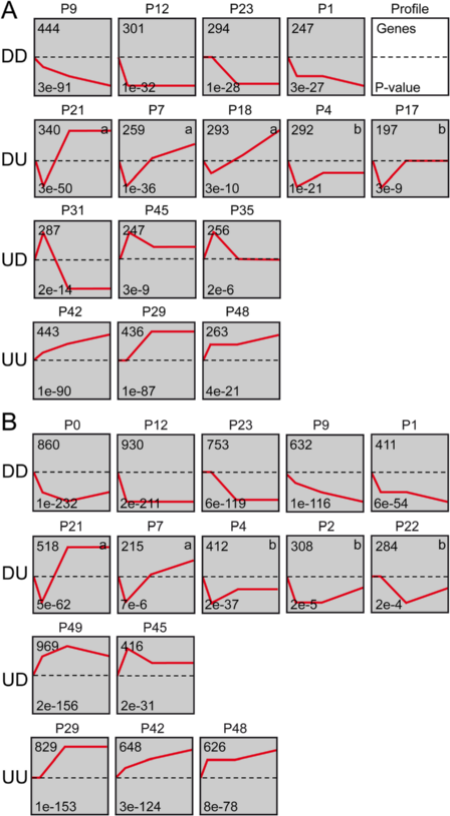
Model profiles identified during AR formation in carnation cultivars. **a** A combined auxin- and mock-treated-dataset is shown for the *2003 R 8* cultivar. **b** The mock-treated dataset for the *2101*–*02 MFR* cultivar. Profiles were classified into four groups: DownDown (DD), DownUp (DU), UpDown (UD), and UpUp (UU) and further ordered based on their P-value (bottom left-hand corner). The number of genes assigned to each profile is shown in the top left-hand corner

As the number of DEGs between auxin-treated and mock-treated samples in the *2003 R 8* cultivar was scarce (see above), we did not distinguish between treatments in this cultivar when performing a GO-enrichment analysis using STEM (see Methods). The expression of 7,341 genes was found to be specifically regulated during AR formation in the *2003 R 8* cultivar and 4,599 of these genes were clustered along different expression profiles and further classified into four major groups based on their expression pattern between 0–6 hAP and 6–54 hAP: DownDown (DD), DownUp (DU), UpDown (UD), and UpUp (UU) (Fig. 4a).

1,286 genes were gradually repressed (DD group) during AR formation in these conditions. Some of the most significantly-enriched GO categories within this group were “response to auxin” (GO:0009733; 15 genes; *P* < 0.001) and “ion transport” (GO:0006811; 46 genes; *P* < 0.001). We assigned 1,381 additional genes to the DU group. About two-thirds of these genes showed an early repression and later became upregulated above their expression at 0 hAP (labelled as “a” in Fig. 4a). The remaining genes in this group, which were quickly downregulated and whose levels were more-or-less restored to initial levels at later time points (labelled as “b” in Fig. 4a), showed enrichment in “photosynthetic membrane” (GO:0034357; 30 genes; *P* < 0.001) encoding genes. Some of the most significantly-enriched GO categories within the DU group as a whole were “cell wall organization or biogenesis” (GO:0071554; 51 genes; *P* < 0.001), “cytoskeleton” (GO:0005856; 46 genes; *P* < 0.001), and “cellular carbohydrate metabolic process” (GO:0044262; 63 genes; *P* < 0.001). Another 790 genes showing a biphasic response were classified into the UD group. Finally, 1,142 genes within the UU group were ranked for GO enrichment: “cell division” (GO:0051301; 32 genes; *P* < 0.001), “microtubule” (GO:0005874; 52 genes; *P* < 0.001), and “cell wall organization or biogenesis” (28 genes; *P* < 0.001) among others. Interestingly, the expression of most genes included within the “cell division” and “cell wall organization or biogenesis” categories peaked after 6 hAP in agreement with the timing of cell cycle re-activation in the cambium observed for this cultivar, as it is shown later.

### Expression profiling in the *2101*–*02 MFR* cultivar and in the response to auxin

12,525 genes were identified in the *2101*–*02 MFR* cultivar as being specifically expressed during AR formation without exogenous auxin treatment (Fig. 4b). 3,586 genes were assigned to the DD group where one of the most significantly-enriched GO categories was “protein serine/threonine kinase activity” (GO:0004674; 175 genes [13.8 % of the protein serine/threonine kinase encoding genes with dynamic expression profiles (EGs)]; *P* < 0.001). 1,737 genes showing a biphasic response were classified into the DU group. Those upregulated at later time points (labelled as "a" in Fig. 4b) encoded proteins enriched in “cytoskeleton” (GO:0005856; 41 genes; *P* < 0.001) and “cell division” (17 genes; *P* < 0.001). Similarly to that found previously for the *2003 R 8* cultivar, the “photosynthetic membrane” (35 genes; *P* < 0.001) category was found enriched among genes whose expression levels were restored to basal levels (labelled as "b" in Fig. 4b). Among the biphasic genes that were assigned to the UD group (1,385), one of the significantly-enriched GO categories found was “carbohydrate derivative metabolic process” (GO:1901135; 52 genes; *P* = 0.002). Finally, 2,103 genes were included within the UU group, where the most significantly-enriched GO categories were “cellular carbohydrate metabolic process” (69 genes; *P* < 0.001) and “cell wall organization or biogenesis” (54 genes; *P* < 0.001). In addition, we found specific GO-enriched categories in profile P29 (Fig. 4b). On the one hand, enriched genes upregulated after 6 hAP (P29) encoded proteins related to “microtubule” (38 genes; *P* < 0.001), “cell division” (24 genes; *P* < 0.001), and “regulation of cell cycle” (GO:0051726; 23 genes; *P* < 0.001). On the other hand, genes encoding putative chromatin-related functions such as “histone H3 lysine 9 methylation” (GO:0051567; 14 genes; *P* < 0.001), or “DNA packaging” (GO:0006323; 14 genes; *P* < 0.001) were also found significantly enriched.

Additionally, the expression of 9,645 genes was found specifically altered during AR formation in the *2101*–*02 MFR* cultivar after exogenous auxin treatment and 5,568 of these genes were significantly clustered to different expression profiles and grouped as described above (data not shown). We found a substantial overlap between EGs of auxin-treated and mock-treated samples (61.2 % for the auxin-treated EGs and 79.5 % for the mock-treated EGs). Consistently, no significant differences in the overall trends of EGs were found between auxin- and mock-treated samples for the *2101*–*02 MFR* cultivar (Additional file 5: Figure S3). However, for a small number of genes assigned to specific profiles in mock-treated samples, we found some changes in their expression profiles after the auxin treatment. About half of the EGs-encoding proteins belonging to “microtubule”, “cell division” and “histone H3 lysine 9 methylation” were reciprocally assigned to profiles P29 (UU) or P21 (DU) in mock-treated and in auxin-treated samples, respectively. We also found that the expression levels of most genes assigned to the “cellular carbohydrate metabolic process” category were complementary at earlier time points in auxin-treated *vs*. mock treated samples.

### Comparative transcriptome profiling of AR formation between carnation cultivars for selected GO categories

**Fig. 5:**
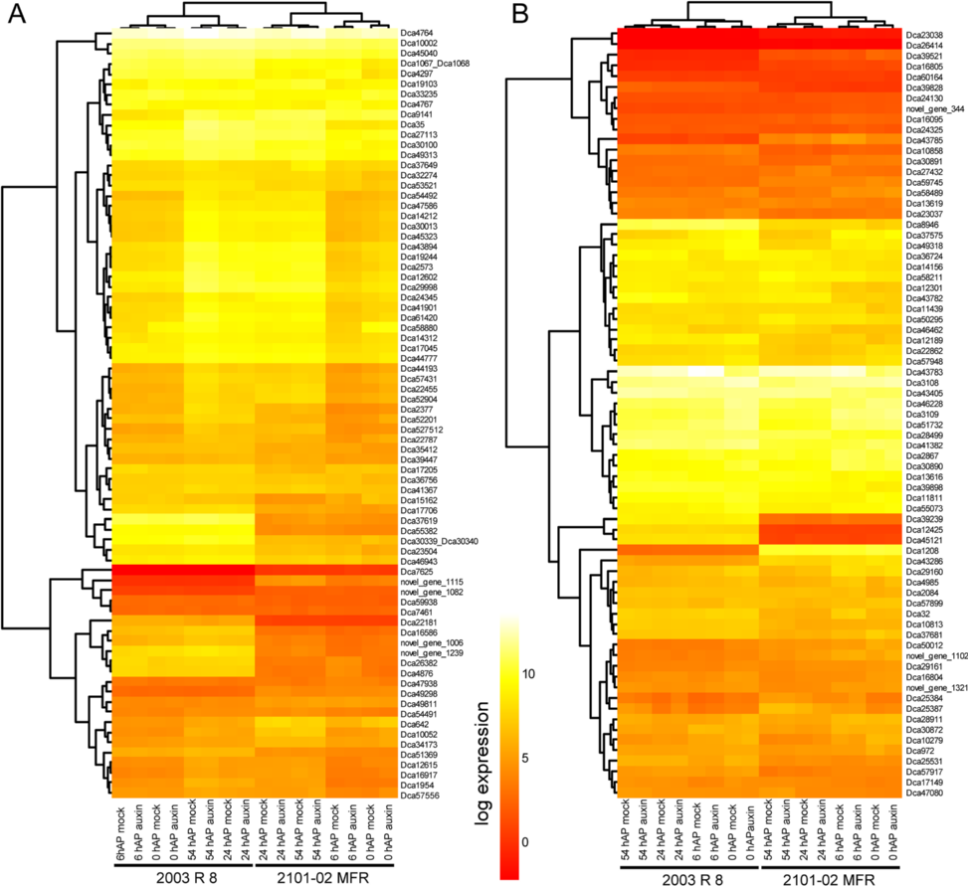
Analysis of expression of transcripts related to cell division (**a**) and response to auxin (**b**) during AR formation. Heat map drawing and clustering was done as described in Methods

To identify genes whose expression correlates with the different stages of AR formation and that could be used as markers, we selected EGs belonging to the GO categories “cell division” and “response to auxin” from the different samples studied (see above). We then built heat map representations from log expression data for all these genes (Fig. 5). On the one hand, the expression of some genes encoding proteins related to cell division were clustered together along the time point series with higher expression at earlier time points, independently of cultivar and treatment (Fig. 5a). Interestingly, genes encoding mitotic cyclins (A-type and B-type) showed a peak of expression between 24 hAP and 54 hAP (Fig. 5a), which is in light with the cellular changes observed in the stem cutting base during AR rooting (see next section). Noteworthy, the majority of these genes moderately respond to the auxin treatment by increasing their expression levels at earlier time points (Fig. 5a). On the other hand, a small number of genes displayed contrasting expression profiles between cultivars. Examples for the latter are the *Dca37619* and *Dca642* genes, which are respectively upregulated and downregulated in *2003 R 8* compared to the *2101*–*02 MFR* cultivar (Fig. 5a).

Considering the expression profiles of genes assigned to the “response to auxin” category, the effect of the auxin treatment was quite small irrespective of the cultivar, and was mainly restricted to earlier time points (Fig. 5b). However, we found striking differences in the expression of a few of these genes between cultivars, such as *Dca1208* and *Dca39239*, which makes them candidates for further studies to analyze their role in the differential response in auxin-mediated AR initiation between these two cultivars.

### Cellular changes in the stem cutting base during AR formation reflects the effect of the auxin signal

**Fig. 6:**
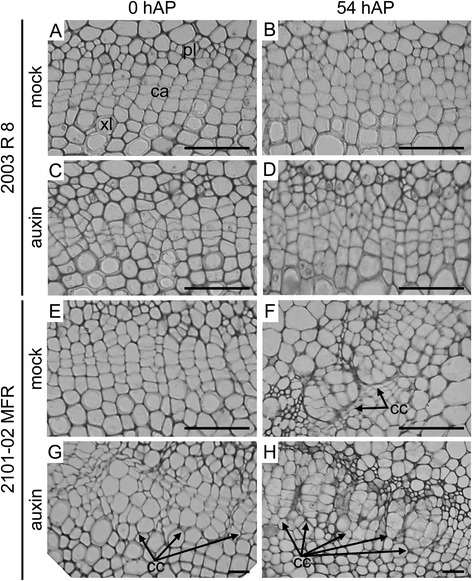
Morphological changes in the ultrastructure of the basal stem of carnation cuttings during adventitious rooting. Light micrographs were taken from cross-sections of stem cutting basal regions in the *2003 R 8* (**a**-**d**) and *2101*–*02 MFR* (**e**-**h**) cultivars, either treated with mock (**a**-**b**, **e**-**f**) or with auxin (**c**-**d**, **g**-**h**) at different time intervals (0 and 54 hAP). ca, cambium; cc, cell clusters; pl, phloem; xl, xylem. Scale bars: 50 μm

In a previous study we found that cell divisions within the cambial region of the stem cutting base took place between 12 hAP and 24 hAP in a good-rooting cultivar used as a reference [43]. We next characterized the cellular changes occurring within the cambium region in the stem cutting base of the *2003 R 8* and *2101*–*02 MFR* cultivars both in mock- and auxin-treated samples to understand the differential responses observed in these two cultivars during AR formation. Although the cambial ring of the *2003 R 8* cultivar displayed a very organized cellular pattern at 0 hAP, we found that some regions within the cambium displayed subtle tissue disorganization (Fig. 6a and Additional file 6: Figure S4). Interestingly, we observed an increase in the number of disorganized regions within the cambial ring at later time points, which could reflect local activation of cell divisions (Fig. 6b). In addition, stem cutting bases of the *2003 R 8* cultivar treated with auxin contained an increased number of these disorganized regions already at 0 hAP (Fig. 6c and 6d). In the *2101*–*02 MFR* cultivar, we observed a higher frequency of small cell clusters within the cambial ring at 0 hAP, which at later time points developed as large clusters of meristematic cells with a disorganized internal structure (Fig. 6f). In most cases, these cell clusters appeared juxtaposed but physically isolated by collapsed neighbouring cells (arrowheads in Fig. 6f). In auxin-treated samples however, cell clusters became apparent already at 0 hAP (Fig. 6g), which is indicative of an early activation of cell division in the *2101*–*02 MFR* cultivar. At 54 hAP, cell clusters were clearly evident and their numbers were higher than in mock-treated samples (Fig. 6h).

To confirm our observations, we estimated some cellular parameters in the two contrasting regions identified within the cambium (Additional file 6: Figure S4; see Methods). In the *2003 R 8* cultivar we observed that the cell division rates significantly differed between organized and disorganized regions at the different time points studied, which seemed not to be affected by the auxin treatment (Additional file 6: Figure S4B). These results suggested that auxin act as a trigger for cell division within a certain population of responsive cambial cells. On the other hand, we observed a significant increase in the number of cells within the cambium at later time points for the *2101*–*02 MFR* cultivar, both in organized and disorganized regions (Additional file 6: Figure S4C), which is indicative of a broad activation of cell division within the cambium, as has been previously described for a good-rooting reference cultivar [43]. Interestingly, the division rate at a given time point was found unchanged irrespectively of the auxin treatment (Additional file 6: Figure S4B). Taken together, these results suggested that auxin acts by promoting divisions of quiescent cambial cells rather than by increasing the number of divisions of already dividing cells, the former producing a net increase in the number of cell clusters within the cambial ring.

### Morphogenetic hormone levels in the stem cutting base during AR formation are correlated with rooting performance

**Fig. 7:**
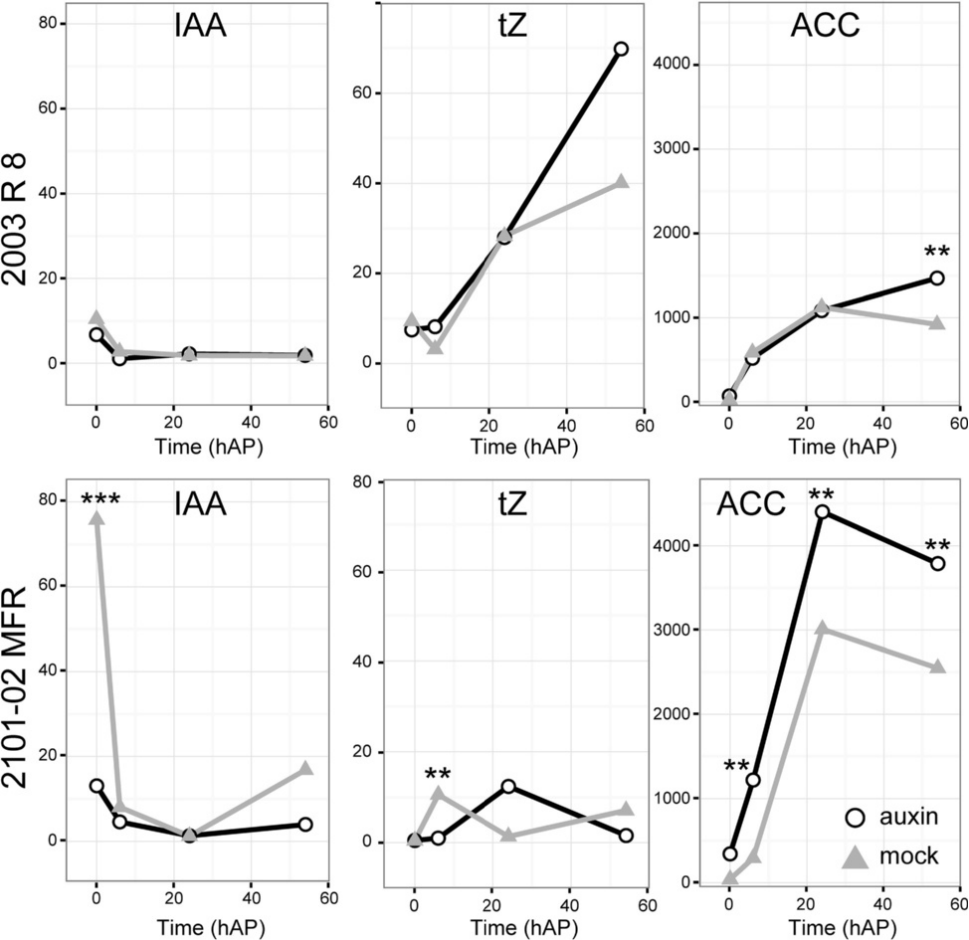
Changes in the concentration of some phytohormones in the basal stem of carnation cuttings during adventitious rooting. Data for indole-3-acetic acid (IAA), *trans*-zeatin (tZ) and the ethylene precursor ACC are shown in ng/g fresh weight. Time refers to hours after planting (0 hAP). Asterisks indicate significant differences (*P* < 0.05) between treatments for a given sampling time

Several plant hormones play a crucial role in controlling AR formation, with auxin and cytokinin playing opposite roles [1, 44]. In addition, wounding stimulates ethylene biosynthesis which is known to positively influence AR formation in some species [45, 46]. We found high levels of endogenous indole-3-acetic acid (IAA) only in the *2101*–*02 MFR* cultivar at 0 hAP, which were quickly downregulated to basal levels (Fig. 7), as previously described [43]. In addition, we found very low levels of *trans*-zeatin (tZ) in the stem cutting base of the *2101*–*02 MFR* cultivar. In contrast, in the *2003 R 8* cultivar, tZ levels steadily increased during the time-course experiment (Fig. 7). Hence, the endogenous auxin/cytokinin ratio estimated as the proportion between IAA and tZ levels was much higher in the *2101*–*02 MFR* cultivar than in the *2003 R 8* cultivar for all time points, with the highest ratio found at 0 hAP. In addition, the levels of the 1-aminocyclopropane-1-carboxylic acid (ACC) ethylene precursor were higher in the *2101*–*02 MFR* cultivar than in the *2003 R 8* cultivar (Fig. 7), which might reflect higher endogenous ethylene production in the *2003 R 8* cultivar.

### TF analysis

**Fig. 8:**
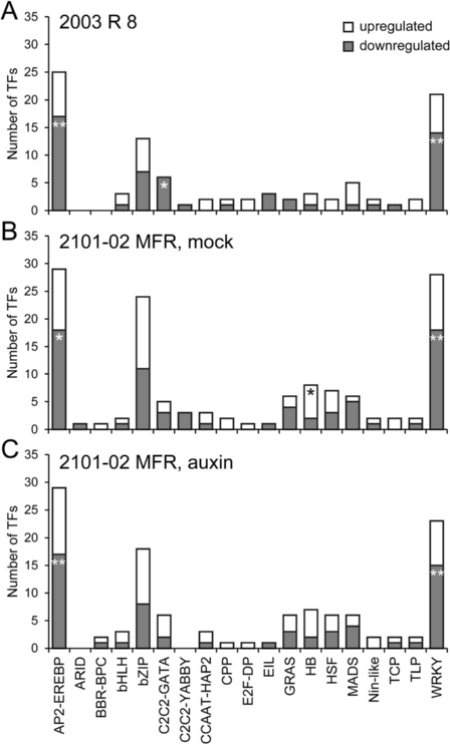
Expression of TF genes during AR formation. The number of upregulated (white bars) and downregulated (grey bars) TFs is indicated for each family in *2003 R 8* (**a**) and *2101*–*02 MFR* mock-treated (**b**) or auxin-treated (**c**) samples. Asterisks indicate significant enrichment of genes (*P* < 0.05) within each TF family

By modulating gene transcription at specific times and during specific processes, TFs and their regulatory networks have important roles in development and stress response. A number of transcripts that show significant expression changes during the time-course experiment performed with STEM, encoded putative transcription factors that belonged to 19 transcription factor families (http://planttfdb.cbi.pku.edu.cn/; Fig. 8). To identify the transcription factors that might regulate the differential rooting responses observed between *2101*–*02 MFR* and *2003 R 8* cultivars, we performed an enrichment analysis using Fisher’s *T*-test (see Methods). In the *2003 R 8* cultivar, a number of genes encoding C2C2-GATA transcription factors, such as *Dca7186* and *Dca56796*, were significantly enriched and showed a clear downregulation of their expression over time (Fig. 8). The WRKY transcription factor family was among the most highly downregulated transcription factor genes irrespective of cultivar and treatment (Fig. 8). Of the 14 carnation genes encoding putative WRKY proteins that were shared between cultivars and treatments, 10 were downregulated over the time-course experiment. Interestingly, the expression of *Dca28099*, the putative carnation ortholog of the Arabidopsis *PLETHORA5* gene [47] was found upregulated only in the *2101*–*02 MFR* cultivar after auxin treatment, which emphasizes its value as an early marker for adventitious root formation in this species.

## Discussion

We followed a next-generation sequencing approach to characterize the gene expression profiles in the stem base of two cultivars with contrasting efficiencies of AR formation and in response to exogenous auxin treatment. It was found that the most significant expression differences were driven by the cultivar, less by the time after planting, and the least by the auxin treatment.

Whereas ARs arise directly from cambial tissues in easy-to-root species such as poplar, callus formation precedes AR initiation in difficult-to-root species such as *Pinus* spp. or *Eucalyptus grandis* [23, 48]. Our histological analysis during root-primordia initiation in two carnation cultivars confirmed that some cambial cells located between the phloem and xylem activate formative (periclinal) divisions in response to the endogenous auxin signal. Next, several clusters of meristematic cells arise along the cambial ring which will later give rise to organized root primordia, as it has been shown previously in the Master reference cultivar [43]. The differences in the rooting ability of the *2003 R 8* and *2101*–*02 MFR* cultivars are due to a delay in the early activation of cell divisions in the former. Exogenous auxin treatment had a similar effect on both cultivars: it accelerated the activation of cell division and it caused a higher number of initials within the cambium. As a result, the rate and the number of ARs increased by the auxin treatment in both cultivars [26], which is in agreement with the inductive effect across plant species of exogenously applied auxins [4]. In addition, the analysis of morphogenetic hormone levels in the two carnation cultivars studied indicated that the bad-rooting behavior of *2003 R 8* was directly correlated with the low ratio of auxin *vs.* cytokinin levels found in this cultivar.

**Fig. 9:**
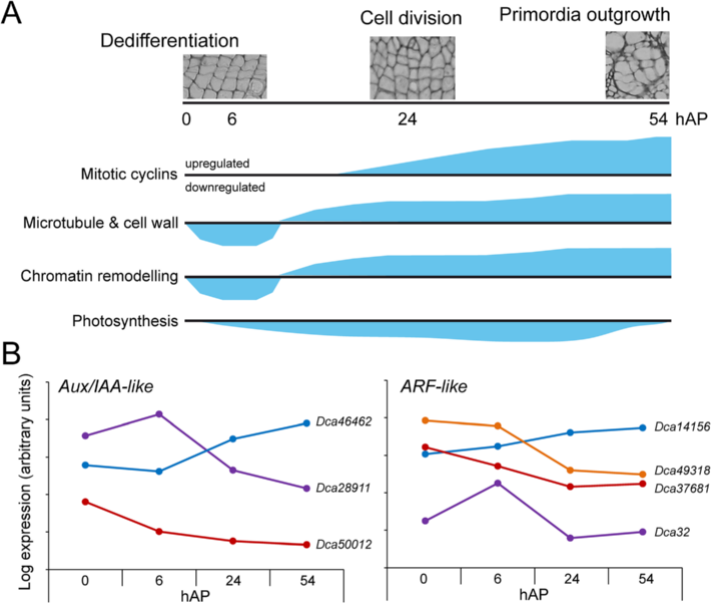
A proposed model of AR formation in carnation stem cuttings derived from transcriptome profiling. **a** Some cellular and molecular events observed during AR formation over the time-course experiment. **b** Representative expression profiles of several genes encoding putative auxin regulators that showed differential expression over the time-course experiment

Consistent with transcriptome data from other species [13, 46], several genes encoding known regulators of auxin response were found differentially regulated during the early stages of AR formation (0 hAP and 6 hAP) in both cultivars. Several EGs encoding putative Aux/IAA corepressors showed specific upregulation 6 hAP in the *2101*–*02 MFR* cultivar and they were found to be transiently induced by the auxin treatment in both cultivars (Fig. 5b). Among them were *Dca28911*, *Dca30890* and *Dca58489*, putative homologs of *SHY2* (also known as *IAA3*) [49], *MASSUGU2* (*IAA19*) [50] and *SOLITARY ROOT* (*IAA14*) [51]. In addition, some other genes encoding Aux/IAA proteins, such as *Dca39239* and *Dca39521*, showed divergent expression levels in the two cultivars studied (Fig. 5b), which suggested that a differential auxin response could explain the differences between good-rooting and bad-rooting cultivars in this species. However, as both cultivars are able to initiate AR formation in response to exogenous auxin, we believe that the auxin response in the *2003 R 8* cultivar is fully functional and the rooting differences observed are due to differential auxin homeostasis between the two cultivars studied. We also found that exogenous auxin treatment did not significantly affect the expression of the EGs encoding putative ARF transcription factors, which also showed similar expression profiles in these two cultivars (Fig. 5b). Our gene expression profiling results are a starting point to identify which auxin response modules involving specific Aux/IAA corepressors and ARF transcription factors are controlling the early steps of AR development in carnation stem cuttings (Fig. 9b).

As previously found during AR development in *Pinus contorta* hypocotyls [21], several integral components of the photosynthetic machinery were downregulated during the initial stages of adventitious rooting and up to 54 hAP (Fig. 9a). This clearly shows that cells within the stem cutting base transiently lack their potential to function as photosynthetic cells, which we believe might be linked to the establishment of a new sink within the stem cutting base, as has been described in petunia cuttings [14]. In line with this hypothesis, we found that the expression of genes encoding sucrose degradation enzymes, such as vacuolar invertase (*Dca8627* and *Dca54544*) and cell-wall localized invertase (*Dca51558* and *Dca59840*), showed a biphasic response during adventitious rooting, coinciding with the onset of the induction phase. In addition, *Dca4507* encoding a homolog of the Arabidopsis SUCROSE SYNTHASE4 [52] was found upregulated after 6 hAP. Our previous results [15, 43] indicated a high energy requirement during rooting in the base of the stem, which was provided by an increase of sucrolytic enzymes during the early phases of rooting. With this study, we confirm that the burst of sucrolytic enzymatic activity observed previously is regulated at the transcript level.

Both the histological analysis and the transcript profiling presented in this work confirmed that the timing for the activation of cell division in the cambial initials depended on the cultivar and it was accelerated by the exogenous auxin treatment. In plants, D‐type and A3‐type cyclins have been implicated in the G1-to-S transition [53, 54] while subgroups of A‐type and B‐type cyclins act in the G2‐to‐M transition [55, 56]. A number of genes encoding mitotic A-type (*Dca24345* and *Dca44777*) and B-type cyclins (*Dca14212*, *Dca43894* and *Dca44193*) were clearly upregulated in both cultivars from 24 hAP onwards and their early expression was slightly higher in the auxin-treated samples. These results are in agreement with those found in Arabidopsis, where the cyclin-dependent kinase activity required for mitosis is regulated by redundant genes encoding CYCLINA2 and CYCLINB [55, 57].

Another of the functional groups that showed differences in their expression levels during rooting were those encoding transcripts related to microtubules (MTs) and MT-associated proteins, such as kinesins. MTs play essential roles in cell division and cell elongation [58] and they indirectly might regulate morphogenesis [59]. Several genes encoding MT-associated proteins showed a biphasic response along the time-course experiment with a clear downregulation at earlier stages and a concomitant upregulation at later time points (Fig. 9a and data not shown). Among those, several kinesin-encoding genes (*Dca24841*, *Dca27864* and *Dca45361*) were found highly expressed from 24 hAP onwards, coinciding with the activation of cell division. Differential remodelling of MTs has been observed previously in juvenile cuttings compared to mature cuttings in *Eucalyptus grandis* [23]. If MT dynamics also plays a role in AR formation in carnation, we expect that subtle perturbations of MTs might improve the rooting success of the bad-rooting carnation cultivars, as it was previously shown for mature *E. grandis* cuttings [23].

Interestingly, we found several genes encoding specific histone variants (*Dca5695*, *Dca16479* and *Dca21788*) that displayed a biphasic expression profile during adventitious rooting consisting of a slight downregulation during the initial stages (up to 6 hAP) and a concomitant increase in their expression levels afterwards. A similar expression profile was found for *Dca58880*, encoding an homolog of the KRYPTONITE (KYP, also known as SUVH4) histone H3 lysine 9 methyltransferase [60]. Recent studies in the *Arabidopsis thaliana* model indicated that H3.3 (whose putative carnation homolog encoding gene was *Dca21788*) was associated with active genes and showed a positive correlation with their expression levels, suggesting that H3 variant replacement may contribute to enable reprogramming at developmental transitions [61]. In addition, the functional loss of KYP resulted in altered expression of developmental regulators, such as *WUSCHEL*, and defects in callus formation during hormone-mediated dedifferentiation [62, 63]. Our results suggest that extensive chromatin remodelling is taking place in the stem cutting base in carnation cuttings prior to the activation of cell division. Whether these chromatin regulators are regulated by the inductive (auxin) signal remains to be elucidated.

## Conclusions

With this work we initiated a multidimensional approach to characterize AR formation in the stem cutting base of a series of carnation cultivars with contrasting rooting performance. Our results allowed us to precisely define the different stages during AR formation and to identify a number of molecular, histological and physiological markers. These will allow us to monitor adventitious rooting in a wide collection of carnation germplasm and to select the good-rooting cultivars for breeding purposes.

### Availability of supporting data

RNA-Seq data supporting this study are available in the ArrayExpress database (www.ebi.ac.uk/arrayexpress) under accession number E-MTAB-3698.

## Additional files

Additional file 1: Figure S1. Exploratory data analysis. (A) Heat map representation of the Euclidean distance between samples. The colour code in the histogram goes from white (lowest correlation values) to dark blue (highest correlation values). (B) PCA analysis. (TIFF 2974 kb) Additional file 2: Table S1. Oligonucleotides used in this work. (DOC 30 kb) Additional file 3:
**Data file for** **evidence-based** **gene models used in this work.** (7Z 1766 kb) Additional file 4: Figure S2. Validation of RNA-seq results by qRT-PCR. The relative expression of six genes was studied in the *2003 R 8* (A) and *2101*–*02 MFR* (B) cultivars. Each dot represents the relative expression data for a given sample. (TIFF 918 kb) Additional file 5: Figure S3. Model profiles comparison between mock-treated and auxin-treated samples in the *2101*–*02 MFR* cultivar. A profile to the immediate left of a yellow bar is from mock-treated samples. A profile to the right of the yellow bar is from the auxin-treated experiment, and has a significant intersection (in terms of the genes assigned to them) with the profile to the left of the yellow bar in its row. The number of genes and the P-value of the intersections are shown in the bottom left-hand. DD, DU, UD and UU are defined in Figure 4. The number on the right, indicates the overlap (in %) between genes assigned to each profile in mock- and auxin-treated samples. Profiles are coloured by default. (TIFF 3893 kb) Additional file 6: Figure S4. Cellular parameters in the cambial cells during AR formation. (A) A representative cross-section image of the stem cutting base used to quantify some cellular parameters within the cambium. Squares represent the area measured for disorganized and organized regions. (B) Division rate of the cambial cells in the studied cultivars. Treatments and time-points are represented by coloured bars (white: mock 0 hAP; light grey: mock 54 hAP; dark grey: auxin 0 hAP; black: auxin 54 h AP). Different letters indicate significant differences (*P* < 0.005) between regions. Error bars indicate the standard deviation (SD) for the mean data shown. (C) Number of cambial cells per mm^2^ in the studied cultivars. Organized and disorganized regions within the cambium are represented as lined and dotted bars, respectively. Asterisks indicate significant differences (*P* < 0.005) between regions at a given time-point. Different letters indicate significant differences (*P* < 0.005) between samples. (TIFF 6084 kb)

## Abbreviations

ACC: 1-aminocyclopropane-1-carboxylic acid
AR: Adventitious root
Arabidopsis: *Arabidopsis thaliana*
ARF: AUXIN RESPONSE FACTOR
bp: base pair
BP: Biological process
CC: Cellular component
cDNA: complementary DNA
DAP: Days after planting
DD: DownDown
DEG: Differentially expressed gene
DNA: Deoxyribonucleic acid
DU: DownUp
EGs: Genes with dynamic expression profiles
GO: Gene ontology
GRETCHEN HAGEN3: GH3
hAP: hours after planting
IAA: indole-3-acetic acid
IBA: indole-3-butyric acid
MF: molecular function
miRNAs: microRNAs
MT: microtubules
NAA: α-naphtalene acetic acid
OBH: one-way best-hits
ORF: open reading frame
PCA: principal-component analysis
qRT-PCR: quantitative reverse-transcription PCR
RNA: ribonucleic acid
RNA-Seq: RNA sequencing
TAIR: The Arabidopsis Information Resource
TF: transcription factor
tZ: *trans*-zeatin
UD: UpDown
UU: UpUp
W/V: Weight/volume
