# Supplementary figures and images for: Gene expression profiling during adventitious root formation in carnation stem cuttings
Source: BMC Genomics. 2015 Oct 14;16:789. doi: 10.1186/s12864-015-2003-5 (PMC4606512; doi:10.1186/s12864-015-2003-5)

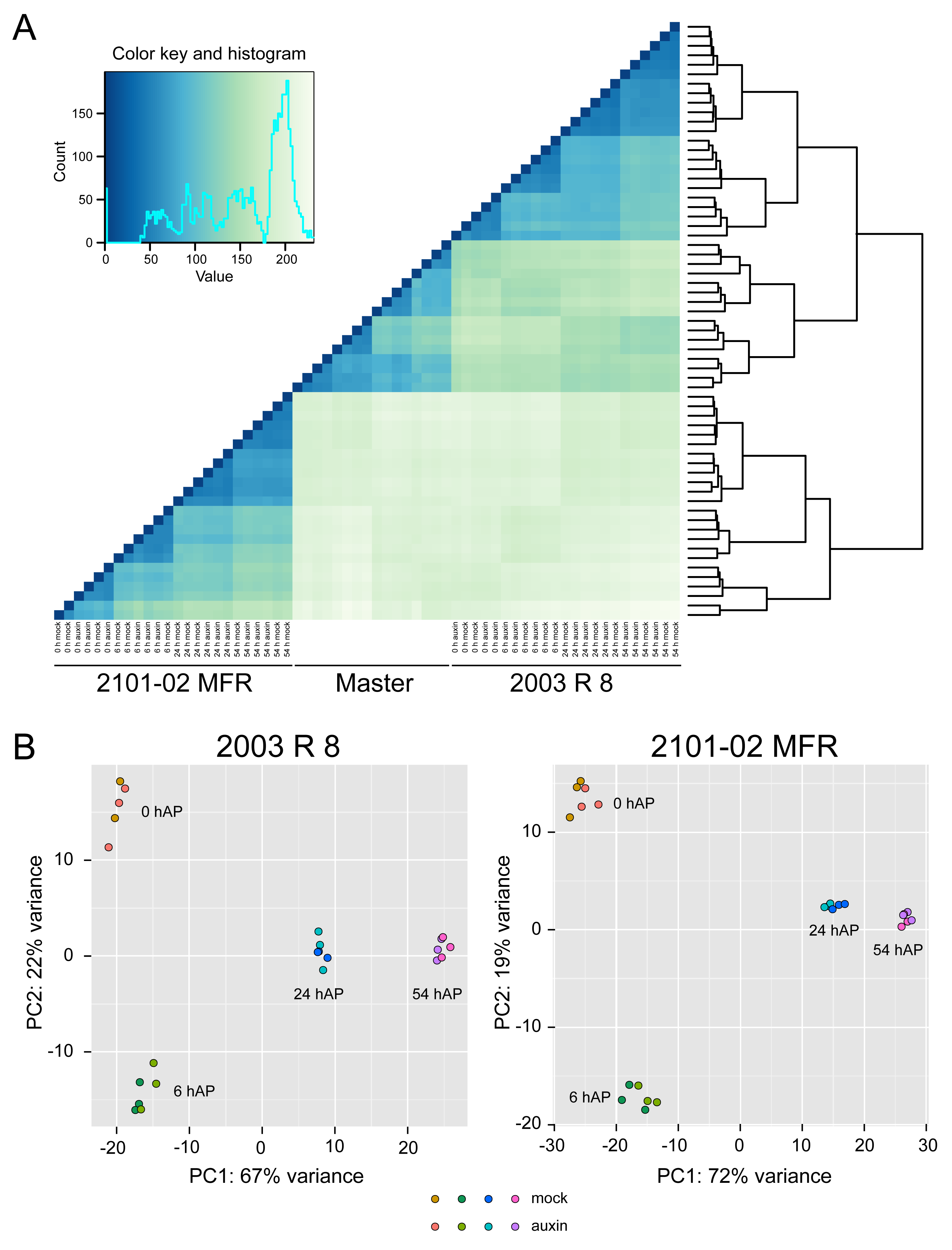

Supplement: Additional file 1: Figure S1. — Exploratory data analysis. (A) Heat map representation of the Euclidean distance between samples. The colour code in the histogram goes from white (lowest correlation values) to dark blue (highest correlation values). (B) PCA analysis. (TIFF 2974 kb) [file 12864_2015_2003_MOESM1_ESM.tif]

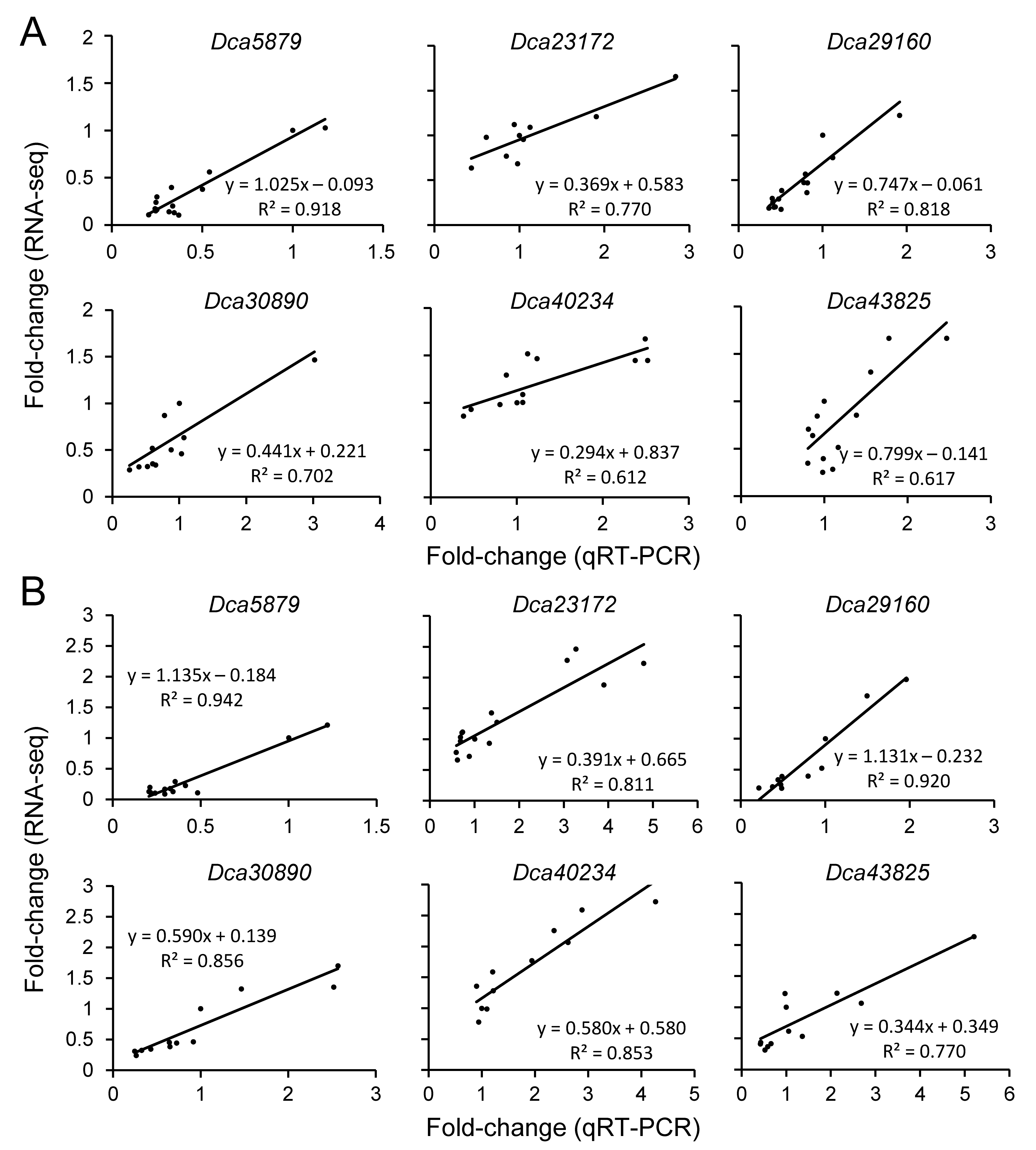

Supplement: Additional file 4: Figure S2. — Validation of RNA-seq results by qRT-PCR. The relative expression of six genes was studied in the 2003 R 8 (A) and 2101–02 MFR (B) cultivars. Each dot represents the relative expression data for a given sample. (TIFF 918 kb) [file 12864_2015_2003_MOESM4_ESM.tif]

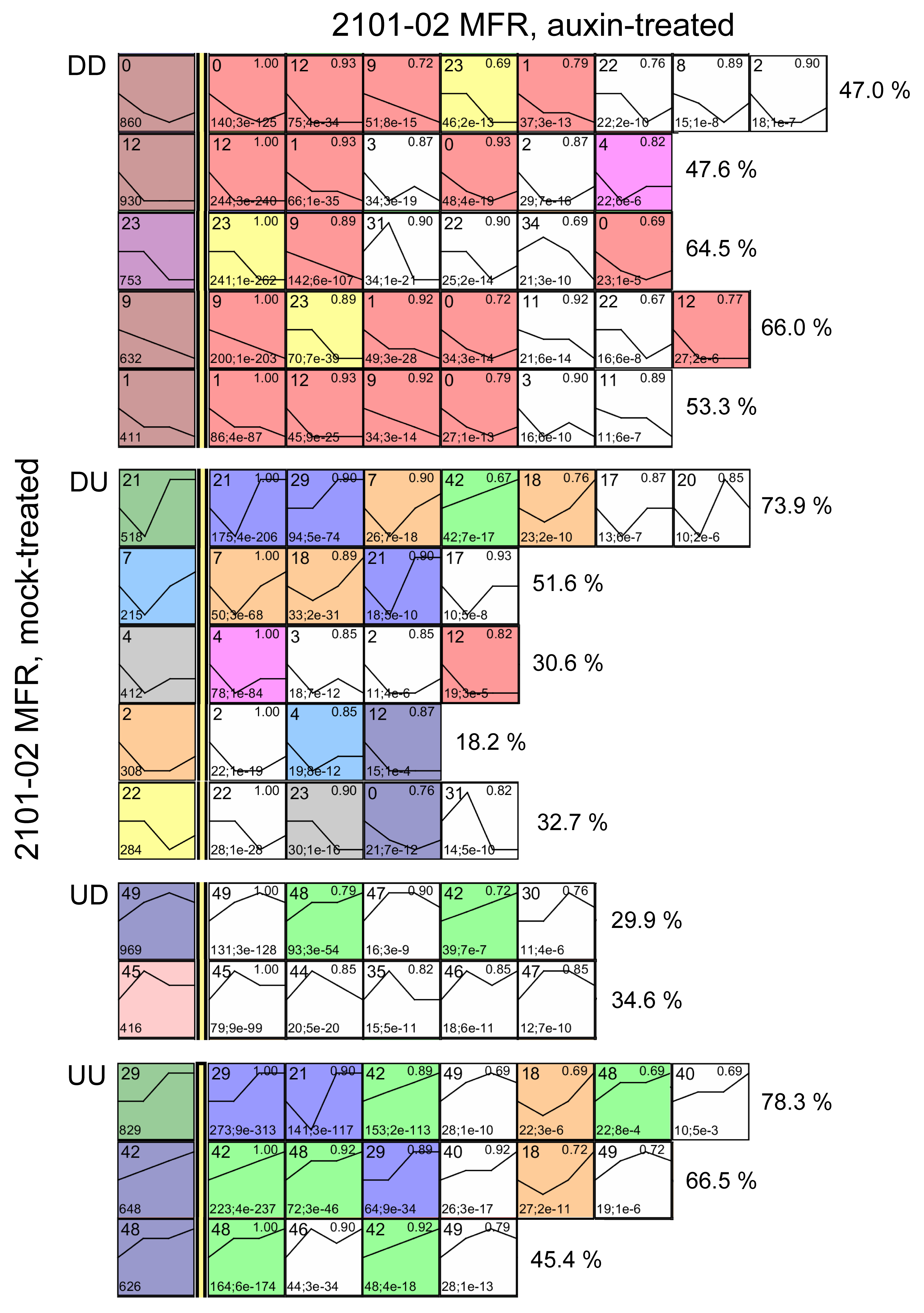

Supplement: Additional file 5: Figure S3. — Model profiles comparison between mock-treated and auxin-treated samples in the 2101–02 MFR cultivar. A profile to the immediate left of a yellow bar is from mock-treated samples. A profile to the right of the yellow bar is from the auxin-treated experiment, and has a significant intersection (in terms of the genes assigned to them) with the profile to the left of the yellow bar in its row. The number of genes and the P-value of the intersections are shown in the bottom left-hand. DD, DU, UD and UU are defined in Figure 4. The number on the right, indicates the overlap (in %) between genes assigned to each profile in mock- and auxin-treated samples. Profiles are coloured by default. (TIFF 3893 kb) [file 12864_2015_2003_MOESM5_ESM.tif]

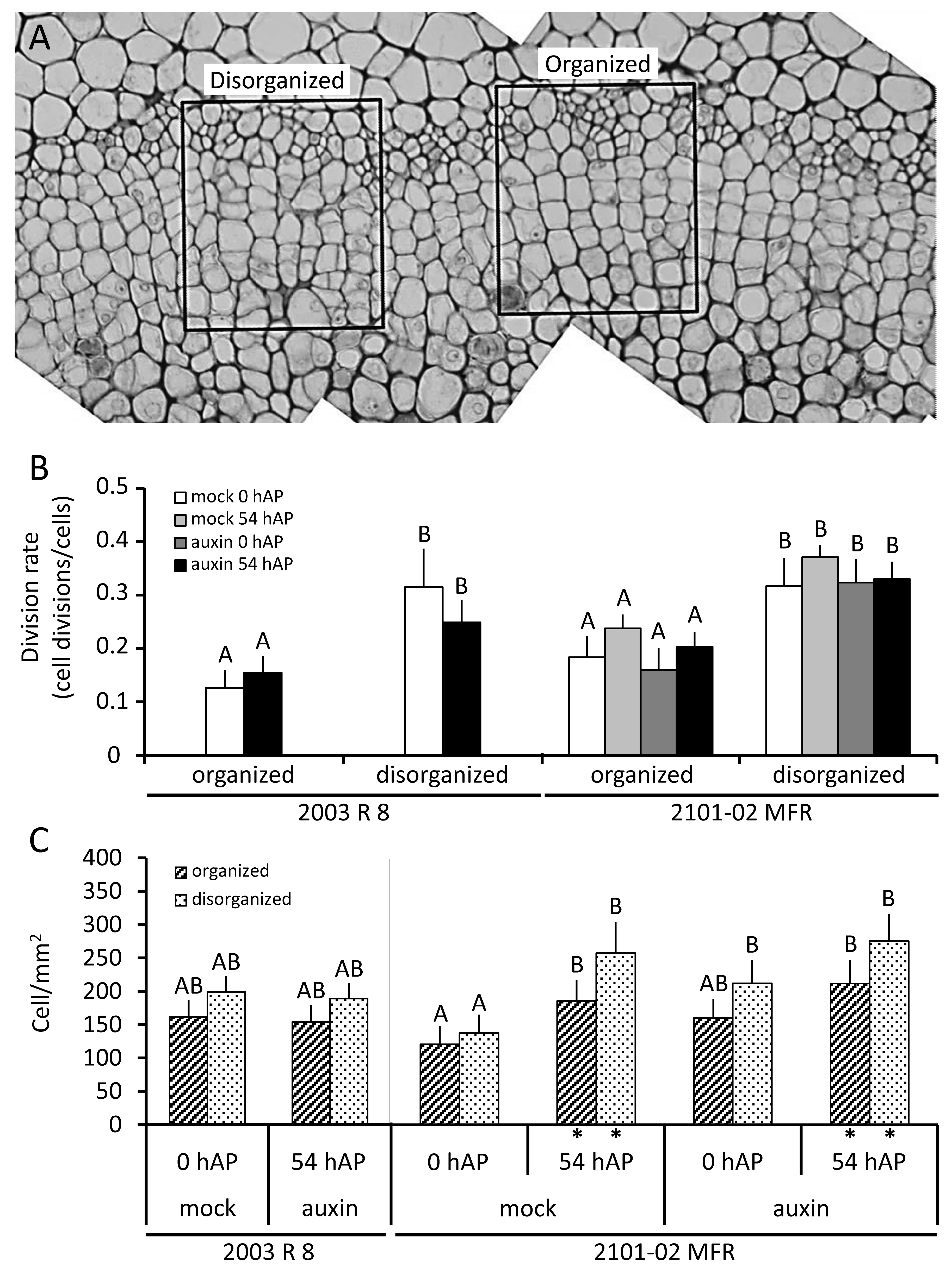

Supplement: Additional file 6: Figure S4. — Cellular parameters in the cambial cells during AR formation. (A) A representative cross-section image of the stem cutting base used to quantify some cellular parameters within the cambium. Squares represent the area measured for disorganized and organized regions. (B) Division rate of the cambial cells in the studied cultivars. Treatments and time-points are represented by coloured bars (white: mock 0 hAP; light grey: mock 54 hAP; dark grey: auxin 0 hAP; black: auxin 54 h AP). Different letters indicate significant differences (P < 0.005) between regions. Error bars indicate the standard deviation (SD) for the mean data shown. (C) Number of cambial cells per mm2 in the studied cultivars. Organized and disorganized regions within the cambium are represented as lined and dotted bars, respectively. Asterisks indicate significant differences (P < 0.005) between regions at a given time-point. Different letters indicate significant differences (P < 0.005) between samples. (TIFF 6084 kb) [file 12864_2015_2003_MOESM6_ESM.tif]
