# Supplementary material for: Gene expression profiling during adventitious root formation in carnation stem cuttings
Source: BMC Genomics. 2015 Oct 14;16:789. doi: 10.1186/s12864-015-2003-5 (PMC4606512; doi:10.1186/s12864-015-2003-5)
Supplement: Additional file 2: Table S1. — Oligonucleotides used in this work. (DOC 30 kb) [file 12864_2015_2003_MOESM2_ESM.doc]

**Table S1.-** Oligonucleotides used in this work

| **Gene locus** | **Oligonucleotide sequences (5’ to 3’)** | | **Product (bp)** |
| --- | --- | --- | --- |
| Dca5879 | TTTCGAGAAACTAGTAGAACAGGCG | GAAGACTGTGTTCTAGCGACAAGCC | 111 |
| Dca23172 | CATTGATCAGCTTTCATTAAGGTTC | CGGTGTTTGCTGTAATTAGAGGCGG | 122 |
| Dca29160 | AGAGCATTACCTGATACGCATGACG | GGCCACCCGACTATTTGTGCCTTGG | 113 |
| Dca30890 | TCATTTTGTTGCTTCTTTTGAGAAG | CATCACCAACAAGGAGCCAATCTCC | 131 |
| Dca40234 | GATGGACTTACCTGTTATAATGCTG | GCTGCCATATGTTTTTAAGCTCCTC | 102 |
| Dca43825 | TTGCCACAATTTAGAAATGCAGTAG | CACCACTCATTACTATGCGACTTGG | 94 |
| Dca17200 | CCAAGCAGCATGAAGATTAAGG | CCTTTGAGATCCACATCTGCTG | 115 |
